# Colorimetric and Longitudinal Analysis of Leukocoria in Recreational Photographs of Children with Retinoblastoma

**DOI:** 10.1371/journal.pone.0076677

**Published:** 2013-10-30

**Authors:** Alireza Abdolvahabi, Brandon W. Taylor, Rebecca L. Holden, Elizabeth V. Shaw, Alex Kentsis, Carlos Rodriguez-Galindo, Shizuo Mukai, Bryan F. Shaw

**Affiliations:** 1 Department of Chemistry and Biochemistry, Baylor University, Waco, Texas, United States of America; 2 Department of Pediatrics, Memorial Sloan-Kettering Cancer Center, New York, New York, United States of America; 3 Department of Pediatric Oncology, Dana-Farber Cancer Institute, Boston, Massachusetts, United States of America; 4 Department of Ophthalmology, Harvard Medical School, Boston, Massachusetts, United States of America; 5 Retina Service, Massachusetts Eye and Ear Infirmary, Boston, Massachusetts, United States of America; Bascom Palmer Eye Institute, University of Miami School of Medicine, United States of America

## Abstract

Retinoblastoma is the most common primary intraocular tumor in children. The first sign that is often reported by parents is the appearance of recurrent *leukocoria* (i.e., “white eye”) in recreational photographs. A quantitative definition or scale of leukocoria – as it appears during recreational photography – has not been established, and the amount of clinical information contained in a leukocoric image (collected by a parent) remains unknown. Moreover, the hypothesis that photographic leukocoria can be a sign of early stage retinoblastoma has not been tested for even a single patient. This study used commercially available software (Adobe Photoshop®) and standard color space conversion algorithms (operable in Microsoft Excel®) to quantify leukocoria in actual “baby pictures” of 9 children with retinoblastoma (that were collected by parents during recreational activities i.e., in nonclinical settings). One particular patient with bilateral retinoblastoma (“Patient Zero”) was photographed >7, 000 times by his parents (who are authors of this study) over three years: from birth, through diagnosis, treatment, and remission. This large set of photographs allowed us to determine the longitudinal and lateral frequency of leukocoria throughout the patient's life. This study establishes: (i) that leukocoria can emerge at a low frequency in early-stage retinoblastoma and increase in frequency during disease progression, but decrease upon disease regression, (ii) that Hue, Saturation and Value (i.e., HSV color space) are suitable metrics for quantifying the intensity of retinoblastoma-linked leukocoria; (iii) that different sets of intraocular retinoblastoma tumors can produce distinct leukocoric reflections; and (iv) the Saturation-Value plane of HSV color space represents a convenient scale for quantifying and classifying pupillary reflections as they appear during recreational photography.

## Introduction

Retinoblastoma (Rb) is an aggressive cancer that forms rapidly in the developing retina of children, typically before the age of five years [1]–[3]. Epidemiology estimates that ∼7000–8000 children develop Rb throughout the world each year, and ∼3000–4000 children die annually [4]. The median age of diagnosis in the U.S. is ∼24 months for unilateral disease and ∼9–12 months for bilateral disease [3], [5]–[7]. Survival rates are high in developed countries (e.g., ∼95% survival in the U.S. [7]–[9]) but drop in resource limited settings (e.g., 48% in India [10]; 46% in Namibia [11], [12]). Lower survival rates are attributed to delayed diagnosis and the development of extra-ocular and metastatic disease [13]. Survivors typically experience moderate to severe vision loss; however, early diagnosis can increase the rate of vision preservation and survival [14]–[18].

**Figure 1:**
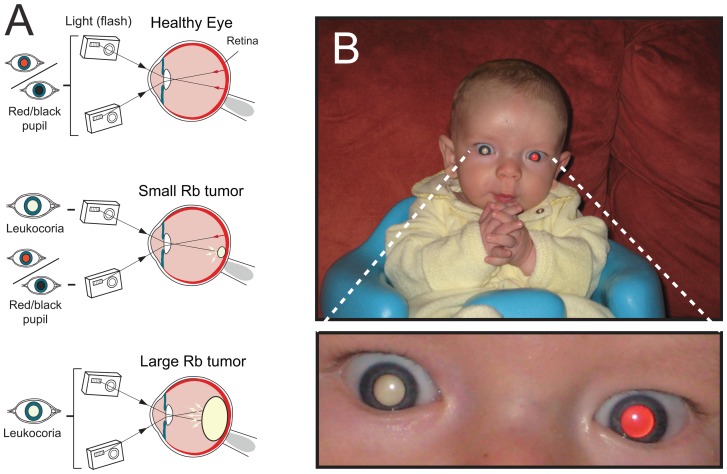
Leukocoria in Children with Retinoblastoma. **A**) The reflection of visible light by an intraocular Rb tumor can cause the pupil to appear white (*leukocoric*) during photography; an increase in the size of a tumor will generally increase the number of photographic angles that will produce leukocoria during recreational photography. **B**) An example of a leukocoric picture from a set of 7377 pictures of a patient (Patient Zero) with bilateral Rb. Images of Patient Zero were donated by his parents.

**Figure 2:**
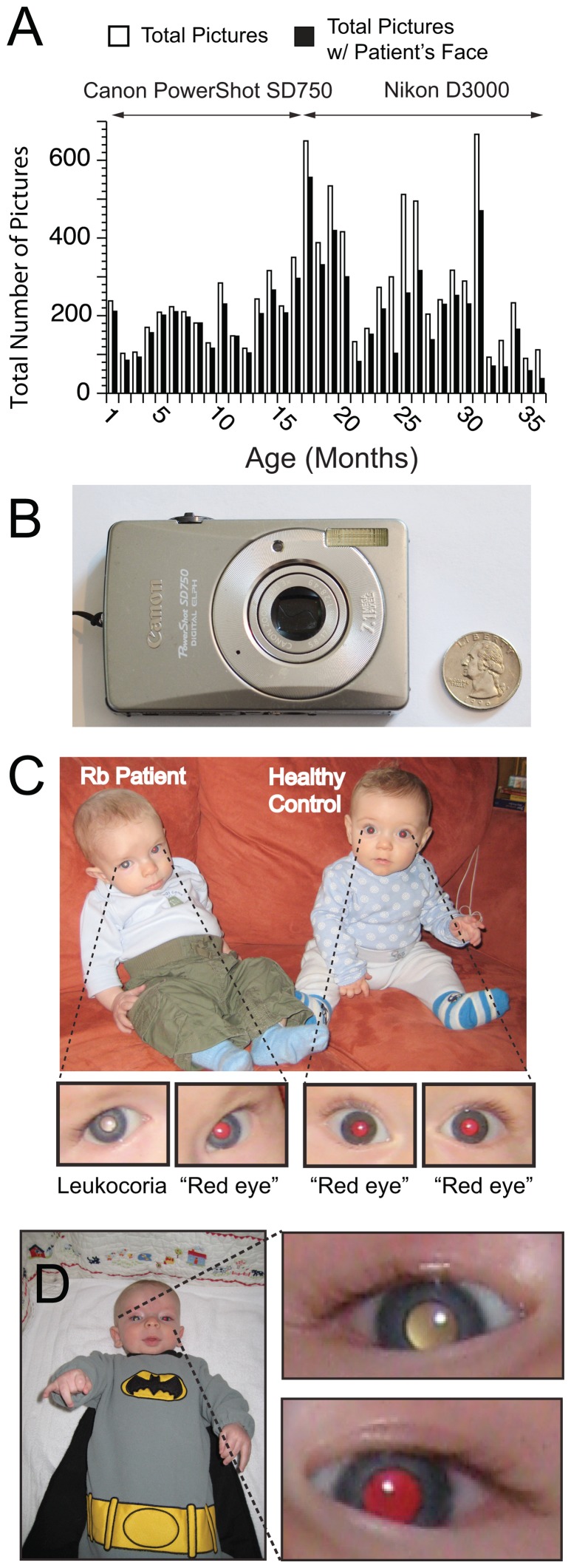
A Collection of ∼7,000 Digital Photographs of a Single Patient with Retinoblastoma. **A**) Longitudinal frequency of photography of “Patient Zero” by parents over a three year period (i.e., from birth to 3 years old; 7377 photographs). **B**) The majority of leukocoric pictures (∼80%) were collected with this compact 7.1 megapixel Canon PowerShot SD750 camera. **C**) Digital picture of Patient Zero (i.e., child on left, exhibiting leukocoria in left eye) and a healthy playmate (i.e., child on right, exhibiting a red reflex in both eyes). **D**) Example of a digital picture of Patient Zero; right eye exhibited leukocoria, and the left eye exhibited a red reflex. Photographs in C & D were taken with Canon PowerShot SD750. Permission to include images of the healthy control child was granted by both parents.

Diagnosing Rb continues to be a major challenge. The incidence of this cancer in the U.S. is sufficiently high (i.e., 1:16,000–18,000 births [7]) that pediatricians are advised to screen for Rb by performing the “red reflex” test with an ophthalmoscope [19], [20]. In spite of pediatric screening, one of the most effective methods for detecting Rb appears to be amateur photography: the diagnosis of a large proportion of Rb cases in the U.S. (e.g., ∼80% in one study [21]) appears to be initiated by a parent's concern over recurrent leukocoria in photographs of their child [22] (see Figures 1B and 2D for examples of Rb-linked leukocoria). Although other rare eye conditions can also result in recurrent leukocoria [23] (e.g., Coats' disease [24], pediatric cataract [25], chorioretinitis [26], and persistent fetal vasculature [27]), the most common cause of *persistent* leukocoria in children under the age of 5 years old is historically considered to be Rb [28]–[30].

Leukocoria (from Greek meaning “white pupil” colloquially referred to as “white eye” or “cat eye”) has been historically associated with advanced Rb and low rates of ocular salvage [21], however, the age of emergence and longitudinal frequency of leukocoria has never been determined for even a single patient. Thus, the correlation between the emergence and frequency of leukocoria (detected by parents during recreational photography) and disease onset, progression and remission remains unknown. We suspect that photographic leukocoria might emerge earlier in disease progression than tacitly assumed, but that it initially occurs at a low frequency because of the small size or eccentric position of the tumor (s), and becomes progressivley more frequent (and thus easily noticed by parents) as tumors increase in size and number.

Despite the effective – albeit, anecdotal – use of digital photography by parents to detect Rb-linked leukocoria [21], there have been no efforts to develop tools that might increase the effectiveness of digital photography in screening Rb (e.g., software that is embedded in a camera or computing device that can detect leukocoria). The recreational photographs of Rb patients with leukocoria have never been analyzed with basic tools in computer graphics that can quantify the colorimetric properties of the leukocoric reflection, e.g., the Hue (color), Saturation (color concentration) and Value (brightness). Thus, a quantitative definition and scale of leukocoria does not exist and the correlation between the clinical severity of Rb (i.e., size, position, and number of tumors) and the colorimetric properties of its leukocoric reflection remains undetermined. We hypothesize that photographs of children with Rb – of the type that parents collect – do contain more clinically relevant information than a simple binary detection of “white-eye”. This information (if present and readily quantifiable) might be useful to a pediatric clinician or ophthalmologist, and could be instantly transmitted out of environments with limited resources, where most deaths occur.

We presume that amateur photography has been overlooked as a quantitative tool for screening Rb because amateur recreational photography involves untrained (or unsuspecting) users who are operating dozens of different devices in diverse settings (i.e., at multiple angles, focal apertures, light intensities, etc.). Nevertheless, despite the optically diverse nature of recreational photography, parents have inarguably proven that this practice of photography is as effective at detecting Rb as pediatric examinations (if not more effective, because parents photograph their children more often than they are examined by a clinician and/or possibly because flash photography involves a rapid flash pulse, t<500 ms, that will not necessarily contract the pupil and impede the reflection of light off peripheral tumors, Figure 1).

In this study, we analyzed >7000 recreational photographs of nine Rb patients and 19 control children (who were photographed alongside patients, i.e., were “playmates”). We show that the intensity of a leukocoric reflection can be quantified in HSV color space; we also show that the lateral and longitudinal frequency of leukocoria can correlate with the clinical severity of Rb and its progression and remission. The results suggests that leukocoria can emerge in the earliest stages of Rb (e.g., at 12 days old in one patient), but occurs initially at low frequency and is, presumably, easily overlooked by a parent. Finally, we propose a quantitative scale by which leukocoria intensity can be graded.

With regard to this study, it must be remembered that the recreational photography of an infant and toddler by his parents is *not* by nature optically random. For example, a parent will typically photograph an infant or toddler at a finite range of focal lengths and will favor certain positions and angles of the child over other angles (i.e., top-down pictures are more often collected than bottom-up). As we show in this paper, thousands of recreational photographs collected over several years can have similar exposure times, focal apertures, and depending upon the camera, a consistent flash pulse and aspect ratio.

## Methods

### Collection of Donated Photographs of Retinoblastoma Patients and Healthy Control Subjects

Photographs of nine children with Rb (2 girls, 7 boys) were donated by their parents. The parents of eight of the children only donated images that they had judged to be leukocoric; these sets of photographs were small (i.e., <10 images per child) and were not longitudinal in nature. The parents of a ninth child (a male, referred to as “Patient Zero”), who are the corresponding authors of this study, donated their entire library of photographs that consisted of an unsorted set of 9493 digital photographs in JPEG (Joint Photographic Experts Group) format. Out of this library, 7377 photographs contained the patient's face and thus were used in analysis. The photographs of Patient Zero also contained images of 19 different children (approximately age-matched) who functioned as embedded controls.

The metadata tags included in the EXIF (Exchangeable Image File) data of each leukocoric JPEG file from each patient were analyzed in order to determine: (i) the date that each picture was collected (i.e., the age of the patient), (ii) whether a flash was used, (iii) whether “Red Eye Reduction” was in effect, (iv) the make and model of the camera, and (v) photographic parameters such as exposure time and focal aperture.

A total of fourteen different digital cameras were used to collect the photographs in this study. Two cameras were used contiguously to collect images of Patient Zero: (i) a Canon PowerShot SD750® (Canon USA, Lake Success, NY) from age 0–16 months, and (ii) a Nikon D3000® (Nikon Inc., Melville, NY) from age 16–36 months. Both cameras were equipped with a xenon flash tube and contained “Red Eye Reduction” and “Red Eye Removal” technologies.

Nine digital cameras that were used to photograph the remaining eight patients were: Apple iPhone 4® (Apple Inc., Cupertino, CA) for Patient 1, n = 9 leukocoric photographs; Panasonic DMC-FS3® (Panasonic Inc., Secaucus, NJ), Canon PowerShot SD300®, and Canon EOS Digital Rebel XSi® for Patient 3, n = 4; Nikon D60® and Blackberry 8330® (Blackberry, Ontario, Canada) for Patient 4, n = 9; Blackberry 8330® for Patient 5, n = 9; Nikon D60® for Patient 6, n = 3; Canon PowerShot A80® and Canon PowerShot A2000 IS® for Patient 7, n = 3; and Panasonic DMC-LZ2® for Patient 8, n = 7. The digital camera used to photograph Patient 2 could not be determined.

The following three digital camera phones were used to generate 72 photographs of a healthy adult that exhibited “pseudo-leukocoria”: Samsung SGH-I997; Apple iPhone 4; and Droid Razr® (Motorola). Because anecdotal evidence suggests that “pseudo-leukocoria” occurs more frequently in low-light conditions, we collected “pseudo-leukocoric” images under low-light conditions (i.e., a dimly lit room) characterized by a light intensity of 0.0259±0.0074 µE/m^2^/s, as measured by a digital light meter (*LX1010B*, Dr. Meter). A flash was emitted during the collection of each “pseudo-leukocoric” image.

### Colorimetric Analysis of Pupillary Reflexes

The average HSV color space parameters (Hue, Saturation, and Value) of each pupil were determined in the following manner: (i) each pupil was cropped in its entirety and the total pixel count was determined using Adobe Photoshop® (Adobe, San Jose, CA; CS5 Extended, version 12.0.4×64); (ii) the number of pixels with a given intensity in three color-channels (red, green, and blue; RGB) was then determined for each pupil; (iii) the average RGB coordinates of each cropped pupil was calculated using Microsoft Excel® (Microsoft Inc., Redmond, WA); (iv) these RGB coordinates were then transformed to the HSV cylindrical coordinate system using the standard RGB-HSV conversion algorithm introduced by Smith [31] (which is operable in Microsoft Excel®). Photographs that contain pupils comprised of 10 pixels or fewer were not analyzed because of their low resolution.

We chose to express and quantify leukocoria in HSV color space (instead of RGB) because HSV specifies the color in terms that are more intuitive (to us) and thus much easier to interpret and communicate. For instance, the HSV system defines a basic color of the visible range of the electromagnetic spectrum (Hue), the concentration of that color, i.e., from pink to red (Saturation), and thirdly, the brightness of the color (Value). The RGB system, on the other hand, partitions a single color into color channels designated “Red”, “Green”, and “Blue” that correspond to the additive color components which make up the single color. While both HSV and RGB can specify a color to the same precision, RGB requires knowledge of additive colors in order to understand how changes in one channel affect the overall appearance of color. Thus, in our opinion, this feature makes RGB more complicated and less intuitive comparing to HSV color space [31]. Moreover, consumers (parents) are often self-educated in HSV color space during the use of electronic image displays (i.e., computer screens, flat screen televisions, etc.).

In the case of Patient Zero, we did not crop and quantify the HSV of *every* pupil in each of the 7377 facial photographs. Instead, we manually inspected each photograph for pupils that were suspicious for leukocoria (i.e., pupils that were not obviously black or dark red in appearance). The entire process of cropping pupils and quantifying HSV parameters for each pupil in this subset was then performed in duplicate by separate researchers.

### Clinical Description of Patient Zero

Patient Zero was diagnosed with bilateral Rb by an ophthalmologist at 123 days of age. The only presenting sign was leukocoria, which the parents had reported noticing for three weeks prior to diagnosis. A diagnosis of Group B disease by the International Classification of Retinoblastoma [5] was made in both eyes, which was based on examination of the dilated eyes and fundus photography. The position and size of tumors were, however, significantly different in each eye. The tumors in the left eye were generally smaller, and although they were posterior with one near the optic nerve and one in the macula (but outside of the fovea), none involved the center of the macula. The right eye contained two tumors: the larger tumor (diameter  = 15 mm) was centrally located, and involved the entirety of the macula; the smaller tumor (diameter  = 1.5 mm) was more peripheral, located at 4 o'clock. The left eye contained three posterior tumors, as described above, with diameters of 6 mm, 1.5 mm, and 0.4 mm.

Over a period of 5 months after diagnosis, Patient Zero received five different types of treatment. In chronological order, the treatments were: (i) systemic vincristine and carboplatin (age: 132–196 days), (ii) focal cryotherapy to right and left eye (age: 200 and 207 days), (iii) focal laser photoablation to right eye (age: 207 days) and left eye (age: 207, 220, 264 days), (iv) enucleation of right eye (age: 220 days, after progression to Group D with vitreous seeding), and (v) proton beam radiation to left eye (age: 222–258 days). Systemic chemotherapy along with cryotherapy and laser consolidation slowed the growth of existing tumors, but failed to reduce their size, and did not prevent the appearance of new tumors, however, treatment with proton beam radiotherapy resulted in an excellent response.

### Ethics Statement

This study was determined to be exempt from review by an Institutional Review Board at Baylor University. The parents of our study participants have given written informed consent, as outlined in the PLOS consent form, to publication of their children's photograph.

## Results and Discussion

We reiterate that the images in this study are photographically diverse (i.e., pictures were collected at multiple photographic angles, poses, settings, and lighting conditions) and thus accurately reflect typical recreational photographs of infants and toddlers in typical recreational activities (i.e., crawling, eating, crying, etc.). This photographic diversity is by no means a limitation or liability to this study – or to the utility of photography in detecting Rb – but rather increases the probability that light will sample the tumor surface and be reflected back towards the camera lens, regardless – to some degree – of tumor position or size (Figure 1A). Moreover, the parents of each child did not anticipate, during photography, that a photograph might be used for a scientific study. The photographs thus represent an authentic set of “family pictures” of the sort that might initiate a diagnosis of Rb, and in the case of Patient Zero, did in fact initiate diagnosis (Figure 1B).

The large number and longitudinal nature of available photographs of Patient Zero allowed us to determine the longitudinal frequency of leukocoria as a function of age and whether the colorimetric properties of leukocoria were statistically different in the right versus left eye. It should be noted that the smaller sets of photographs of the other eight patients are not longitudinal in nature, or large enough (in our opinion) to justify a statistically significant comparison between leukocoria intensity and clinical severity, but are useful for surveying the possible range of Rb-linked leukocoria in HSV color space.

### Longitudinal Frequency of Leukocoria in “Patient Zero”: From Birth through Diagnosis and Remission

The longitudinal frequency of photographs of Patient Zero is shown in Figure 2A. The parents collected photographs consistently over a period of three years. We manually analyzed this entire set of photographs and found that 237 out of 7377 pictures contained at least one leukocoric pupil; leukocoria was detected in 120 left pupils and 146 right pupils. Approximately 80% of the leukocoric pictures were taken with a Canon PowerShot SD750 (shown in Figure 2B). A pupil was classified as leukocoric if it exhibited an abnormal reflection with a Value ≥0.50, and a Saturation that was ≤0.60 (in HSV color space). Approximately 10% of pupils that were categorized as leukocoric exhibited an average pixel Value ≤0.5 or Saturation ≥0.60, but were nonetheless classified as leukocoric because only a portion of the pupil exhibited abnormal Saturation or Value. In contrast, many non-leukocoric pupils (from Patient Zero and control subjects) contained a specular reflection of the cornea (which is not indicative of disease) that caused the average pixel brightness to be >0.5. This type of specular reflection is common in flash photography and appears as a white dot in the pupil, iris or sclera. We did not attempt to subtract specular reflections from images of any patient or control subjects because our goal is to determine how effective digital photography – as practiced by amateurs during recreation – can be at quantifying leukocoria.

**Figure 3:**
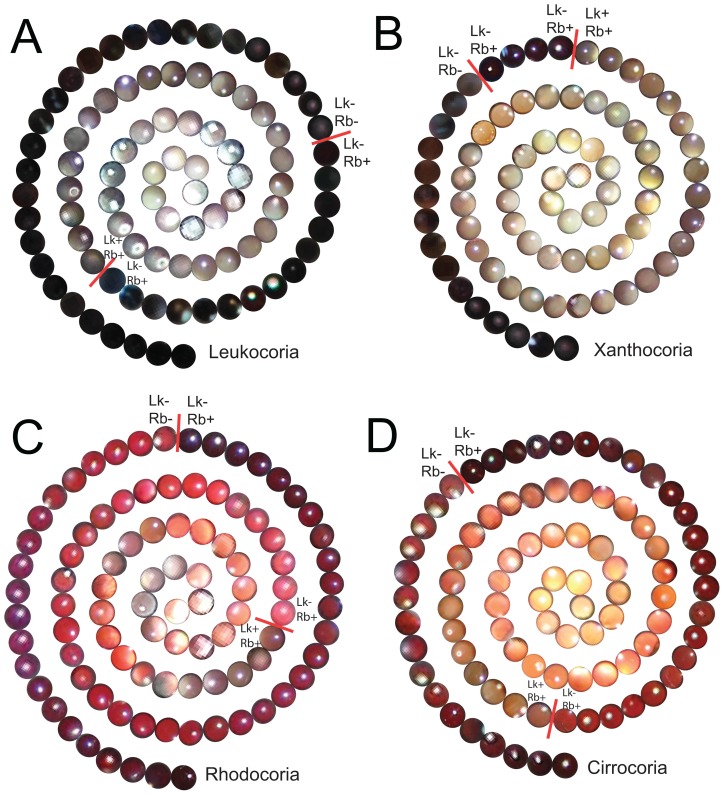
Examples of Cropped Leukocoric and Non-Leukocoric Pupils from a Set of 7377 Pictures of Patient Zero (and Control Children Who Were Photographed Alongside Patient). Each spiral contains: (i) cropped leukocoric pictures from Patient Zero (denoted Lk+/Rb+), (ii) non-leukocoric pupils from Patient Zero (Lk−/Rb+), and (iii) non-leukocoric pupils from healthy control subjects (Lk−/Rb−). **A**) Cropped leukocoric pupils that exhibit a gray scale (classic leukocoria); cropped leukocoric pupils with non-black and white appearance are also shown: **B**) yellow, i.e., “xanthocoria”; **C**) pink, i.e., “rhodocoria”; **D**) orange, i.e., “cirrocoria”. Many pupils in A–D contain specular reflections of cornea that appear as a white dot and are not indicative of disease.

Examples of leukocoria from the donated set of photographs of Patient Zero are shown in Figures 1B and 2D. In addition, approximately 300 cropped images of pupils from Patient Zero and healthy control subjects are grouped according to gross shade and arranged into spirals (Figure 3). Each spiral contains: (i) cropped pupils of Patient Zero that exhibited leukocoria (denoted “Lk+/Rb+”); (ii) non-leukocoric pupils from the patient (which appear black or red, denoted “Lk-/Rb+”); and (iii) cropped pupils from healthy subjects that appeared red or black (denoted “Lk-/Rb-” in Figure 3). Leukocoric pupillary reflections were not detected in healthy control subjects (however, leukocoria can occur rarely in children who do not have any known eye disease, presumably during off-axis photography and reflection of the optic nerve [32], [33]).

The gross appearance of leukocoric pupillary reflections in Patient Zero was often white or gray (Figure 3A), but leukocoria also appeared with yellow Hues (Figure 3B), pink Hues (Figure 3C) and orange Hues (Figure 3D). The photographic reflection of Rb tumors might, therefore, be more accurately described by a general term such as “photocoria” (Greek: light pupil), instead of leukocoria, because the abnormal reflections do not necessarily *appear* white [34]. We attribute the differences in the gross appearance of photocoric pupils to be caused by different angles of photography, which result in variable mixtures of light reflected from the healthy regions of the retina and optic nerve, and light reflected by the surface of a tumor.

**Figure 4:**
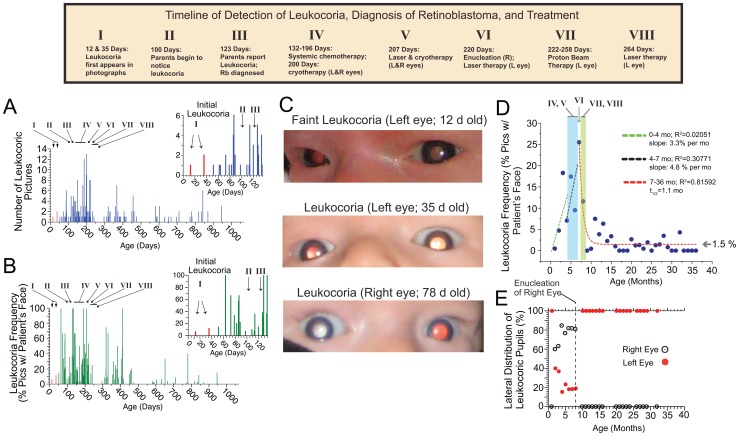
Comparison of Frequency of Leukocoria with Age of Patient Zero and Timeline of his Treatment. **A**) Number of leukocoric pictures plotted as a function of age. Inset shows expansion of age 0–135 days. **B**) Daily frequency of leukocoric pictures from a set of 7377 facial pictures plotted as a function of age. Inset shows expansion of age 0–135 days. **C**) First leukocoric pictures of patient at 12, 35, and 78 days old. **D**) Comparison of monthly frequency of leukocoria with treatment of patient. E) Lateral distribution of leukocoria in 7377 photographs of Patient Zero. After the first month of life, the right eye accounted for the majority of leukocoric pupils that were observed until the right eye was enucleated.

A timeline of the diagnosis, treatment, and remission of the Patient Zero is described in Figure 4, and compared with the daily and monthly frequency of leukocoria. Leukocoria first occurred at 12 days old (Figure 4A–C) – several months before the parents first noticed leukocoria – but only occurred in <5% of facial pictures taken during the first month of life (Figures 4B,4D). Leukocoria increased in frequency during disease progression (reaching as high as 100% of pictures per day and 25% of pictures taken per month, Figures 4B,4D), even in spite of systemic chemotherapy, laser photoablation therapy, and cryotherapy. The increase in frequency, despite chemotherapy, is consistent with clinical observations that systemic chemotherapy did not significantly reduce tumor size, or prevent the formation of new small tumors (which were immediately and successfully treated with cryotherapy or laser photoablation therapy). The treatment of the patient's left eye with proton beam radiation and laser photoablation (which resulted in long term tumor regression) decreased the frequency of leukocoria to <2% per month (Figure 4D). Leukocoria frequency remained <2% per month throughout the period of remission.

The lateral distribution of leukocoria in Patient Zero is shown in Figure 4E. The right eye accounted for 60–85% of all detected leukocoria (until it was enucleated at 9 months of age). We attribute this higher frequency to the greater total surface area of tumors in the right eye and their central location, which might increase the probability (during recreational photography at multiple angles) that light will reflect off the surface and into the camera lens (Figure 1A). The total surface area of tumors in the right eye was calculated to be ∼4-fold greater than the surface area of tumors in the left eye. The lateral ratio of the total surface area of tumors in the right and left eye was approximated using the measured height and diameter of tumors from fundus photography performed at age 129 days, and 199 days; a semi-spherical geometry was assumed when calculating the surface area of each tumor, as previously described [35]. The correlation between the frequency of leukocoria and the progression and remission of disease and also the greater frequency in the more severely affected eye suggests that the leukocoria observed in these images are clinically relevant, and that leukocoria frequency can be a clinically relevant parameter.

### Right Leukocoric Pupils of Patient Zero Exhibited Lower Saturation and Value than Left Pupils

**Figure 5:**
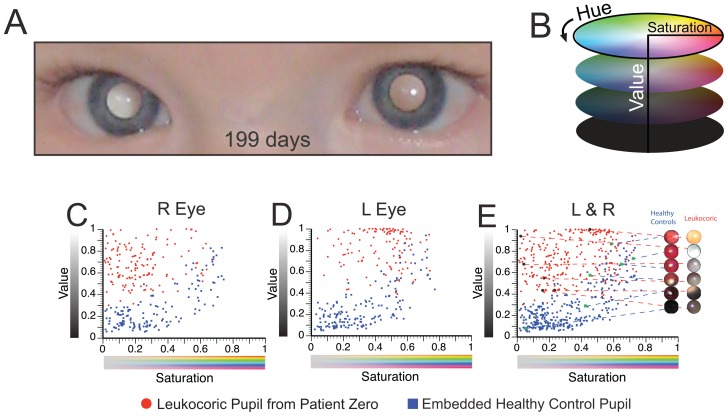
Quantification of Saturation and Value of Right and Left Leukocoric Pupils of Patient Zero and 19 Healthy Control Children. **A**) Digital image showing bilateral leukocoria in Patient Zero taken at the age of 199 days. **B**) Illustration of cylindrical HSV (Hue, Saturation, Value) color space. **C**) Plot of average Saturation and Value of cropped leukocoric and control pupils from right eyes of Patient Zero (red circles) and 19 control subjects (blue squares). **D**) Plot of average Saturation and Value of cropped leukocoric and control pupils from left eyes of patient (red circles) and 19 control subjects (blue squares). **E**) Saturation and Value from right and left leukocoric and control pupils (a combination of plots C and D). Images of cropped pupils are matched to enlarged data points in order to illustrate the range of Saturation and Value of leukocoric and control pupils.

**Figure 6:**
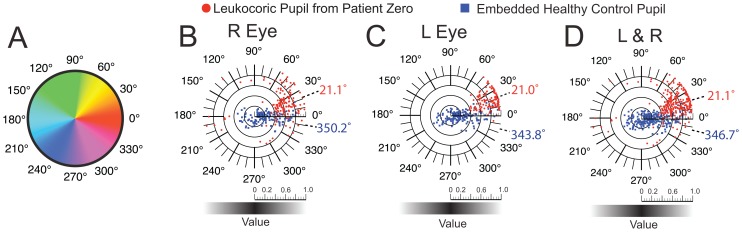
Quantification of Hue and Value of right and left Leukocoric Pupils of Patient Zero and 19 Healthy Control Children. **A**) Depiction of Hue as an angular quantity. **B**) Polar plots of average Hue, per pixel (angular dimension) and average Value, per pixel (radial dimension) for right eye of patient that exhibited leukocoria (red circles), and right eye from 19 healthy children (blue squares). **C**) Polar plots of average Hue, per pixel (angular dimension) and average Value, per pixel (radial dimension) for left eye of patient that exhibited leukocoria (red circles), and left eye from 19 healthy children (blue squares). **D**) Combination of data points from plots C and D.

The Saturation and Value of the right and left leukocoric pupils from Patient Zero are plotted in Figure 5 (as a per-pixel average, red circles). The average Hue versus Value of each leukocoric pupil is also plotted on a polar coordinate plane (Figure 6, red circles). Because the HSV quantities are expressed as a per-pixel average (the average number of pixels analyzed was 308.11), they are independent of image resolution. We also calculated the mean Hue, Saturation, and Value of right and left leukocoric pupils over the entire three-year period of photography (Table 1). These colorimetric (and statistical) analyses of pupils demonstrate that the Saturation and Value, but not the Hue of right leukocoric pupils, are different than left leukocoric pupils in Patient Zero. For example, the three-year aggregate mean Saturation of right leukocoric (RL) pupils (S_RL_ = 0.234) is 46% lower than left leukocoric (LL) pupils (S_LL_ = 0.436; p<0.0001^*^). The aggregate mean Value of the right leukocoric pupils (V_RL_ = 0.677) is 17% lower than left pupils (V_LL_ = 0.818; p<0.0001^*^). The right and left pupils did not show differences in Hue: the three-year aggregate mean Hue of right leukocoric pupils (H_RL_ = 21.1°, i.e., yellow) were nearly identical to left leukocoric pupils (H_LL_ = 21.0°). The derivation of the p values and statistical significance of differences in the HSV quantities of right and left eyes are discussed below.

**Table 1:** Mean HSV Quantities of Leukocoria in “Patient Zero” and Control Pupils Over 3 Years.

	Leukocoric, left; n = 120	Leukocoric, right; n = 146	Control, left; n = 166	Control, right; n = 139
Huea,d	21.0° (0.033°)	21.1° (0.215°)	343.8° (1.060°)	350.2° (0.877°)
Saturation^b^	0.436 (0.159)	0.234 (0.166)	0.317 (0.194)	0.322 (0.216)
Value^c^	0.818 (0.168)	0.677 (0.166)	0.280 (0.210)	0.297 (0.216)

a For Hue of R and L non-leukocoric controls, p>0.05; for Hue of R and L leukocoric pupils, p<0.05*; p-values for Hue were calculated with Wheeler-Watson test.

b For Saturation of R and L controls p = 0.9845; for Saturation of right and left leukocoric pupils p<0.0001*.

c For Value of R and L controls, p = 0.4508; for Value of R and L leukocoric pupils p<0.0001*.

d Error values in parentheses are standard deviation, except for those of Hue, which are circular standard deviation (CSD). CSD is a circular statistical analogue of standard deviation which measures the spread of the data points about the average center.

The ability to detect variations in the average colorimetric properties of leukocoric reflection from different ocular sets of Rb tumors with a pocket-sized digital camera (Figure 2B), during recreational photography is remarkable. We hypothesize that the lower Saturation of leukocoric reflections from the right eye, compared to the left for Patient Zero, is caused by the greater degree of retinal eclipsing by the larger surface area of the tumors in the right eye compared to the left eye.

### Right and Left Pupils are Colorimetrically Identical in Healthy Children

It is possible that the bilateral differences in Saturation and Value of leukocoria in Patient Zero resulted from a photographic clustering artifact, i.e., images were collected at a constant angle, lighting, pose, or setting which may lead to a measurable amount of clinically irrelevant leukocoria (i.e., pseudo-leukocoria [32]). To begin to rule out this possibility and to establish quantitative and colorimetric definitions of healthy pupillary reflexes, we measured the Hue, Saturation, and Value of right and left pupils from 19 healthy children (without having any known eye diseases; 305 pupils in total; 166 left pupils; 139 right pupils; mean age  = 39.5 months old, median age  = 20 months old, as adjusted to their frequency of appearance alongside Patient Zero in photographs). The images of these children represent a convenient set of internal controls from which we could determine the average HSV in healthy pupillary reflections and also further ascertain if the two cameras used resulted in high levels of clinically irrelevant “pseudo-leukocoria” (caused, for example, by reflection of the optic nerve). As an instance, each control child was – by virtue of being photographed alongside Patient Zero – also photographed with the same camera as Patient Zero, under the same lighting conditions, exposure time, flash pulse duration, and aperture (Figure 2C). The HSV were determined for pupils from each healthy child in the same manner as leukocoric pictures and regardless of the gross appearance of the healthy child's pupil. The colorimetric properties of control pupils should be identical among the right and left eyes of these children, so long as no photographic clustering artifact (pseudo-leukocoria) is present in these data.

Plots of the Saturation and Value of right and left control pupils are shown in Figure 5 (blue squares). Polar plots of Hue (angular) and Value (radial) of right and left control pupils are shown in Figure 6 (blue squares). The aggregate mean Hue, Saturation and Value for all 139 right and 166 left control pupils that were photographed over the three-year period are listed in Table 1. These quantities represent a reasonable starting point for establishing standard colorimetric properties of pupillary reflexes of healthy children (at the age of Rb susceptibility) during digital photography.

The mean Hue, Saturation, and Value for all right control pupils were nearly identical to those of the left control pupils. For example, the mean Hue of right control (RC) pupils (H_RC_ = 350.2°) differed only 6.4° from left control (LC) pupils (H_LC_ = 343.8°; p>0.05); the bilateral Saturation differed by only 2% (p = 0.9845) and the Value by 6% (p = 0.4508). The similarities in the HSV of right and left control pupils suggest that: (i) leukocoria detected in this study is only observed in a patient with Rb and thus is clinically relevant, (ii) the cropped pupils from 19 different control subjects have similar colorimetric properties (Table 1), and most importantly, (iii) any differences that are detected in HSV quantities of right and left leukocoric pupils from Patient Zero are not caused by photographic clustering artifacts, but are instead, caused by clinical differences in each eye.

### Statistical Significance of Bilateral and Longitudinal Differences in Hue, Saturation, and Value of Cropped Pupils from Patient Zero and Healthy Control Subjects

In order to determine if the Saturation and Value of each set of right and left cropped pupils (from Patient Zero and healthy control subjects) were normally distributed, we performed a Shapiro-Wilk test. We did not perform a similar statistical analysis on photographs of other patients with Rb because of the small number of photographs (i.e., <10) of each child.

The results of the Shapiro-Wilk test demonstrated that the quantities of both Saturation and Value of right and left pupils were characterized by a non-normal distribution (p<0.005^*^). The absence of a normal distribution demonstrates that a non-parametric statistical test (e.g., the Van der Waerden test) is most appropriate to compare the statistical similarity of the Saturation or Value of cropped pupils from each eye of the patient and control subjects. We therefore used the Van der Waerden test to determine p-values of Saturation and Value between right and left pupils, and to determine if the Saturation of right leukocoric pupils is associated with the same mathematical distribution as Saturation of left leukocoric pupils. The results demonstrate that the differences in Saturation and Value of right and left pupils from Patient Zero are statistically significant (Table 1).

In order to determine if the differences in the Hue of right and left pupils were statistically significant, we used the Wheeler-Watson test. Because the Hue of cropped pupils is expressed as a directional (circular) statistic, the Shapiro-Wilk test for normality and the Van der Waerden test – which were designed for use on non-directional data – are not applicable. The Wheeler-Watson test is a non-parametric test designed to determine statistical similarity between the distributions of different sets of directional data, and is thus appropriate for comparing Hue of right and left eyes, etc. The Hue of right and left pupils were not signifantly different (Table 1).

### The Average Hue of Leukocoria in Patient Zero is Yellow

The three-year mean Hue of right and left leukocoric pupils of Patient Zero exhibited a yellow Hue, in comparison to right and left pupils from control subjects, which exhibited a red Hue (Figure 6). We hypothesize – but cannot prove – that the yellow Hue associated with this patient's leukocoria resulted from the chemical composition and/or surface properties of the Rb tumor. While this hypothesis is bold, it is by no means capricious. For example, the diverse chemical composition of the *tapetum lucidum* (e.g., guanine, collagen, or riboflavin) among nocturnal animals is thought to cause the variably colored eye-shines (i.e., retinal reflexes) that are commonly observed among these animals (ranging from blue in bovine to yellow-green in canine) [36]. The *tapetum lucidum* is a reflective layer of retinal tissue (not present in humans) that functions as a biologic reflector system to enhance visual sensitivity in low-light conditions [36].

Previous analyses of Rb tumors from both fundus photography and pathological analyses of surgical specimens from enucleated eyes show that Rb tumors can be white, “off-white”, tan, or yellow in appearance [37]. Rb tumors (or regions of tumors) that are yellow have been associated with hemorrhage, macular yellow pigment, calcification, and necrosis [37], however, it is possible that the yellow color we detect arises from the lipid composition of the plasma membrane of tumor cells. The lipid constituents of Rb cells have not been determined exactly and categorically, and the lipid content of cultured Rb cells can vary among different *Rb^−^/Rb^−^* cell lines [38]. Retinoblastoma tumors have been reported to possess increased levels of unsaturated fatty acids [39]–[42], as well as a higher content of cholesterol than healthy cells in the retina [43]. Intraocular cholesterosis (abnormal deposition of cholesterol) has also been reported in children with Rb after systemic chemotherapy, cryotherapy and laser photoablation [44]. No clinical deposition of cholesterol (e.g., hard exudation) was observed in the eyes of Patient Zero. Nevertheless, the degree to which cholesterosis occurred in Patient Zero, at any time throughout his lifetime, is unknown and thus we can only speculate on the cause(s) of the yellow Hue.

### Saturation and Value of Right Pupils from Patient Zero Remain Different From Left Pupils throughout Three-Year Period of Treatment

**Figure 7:**
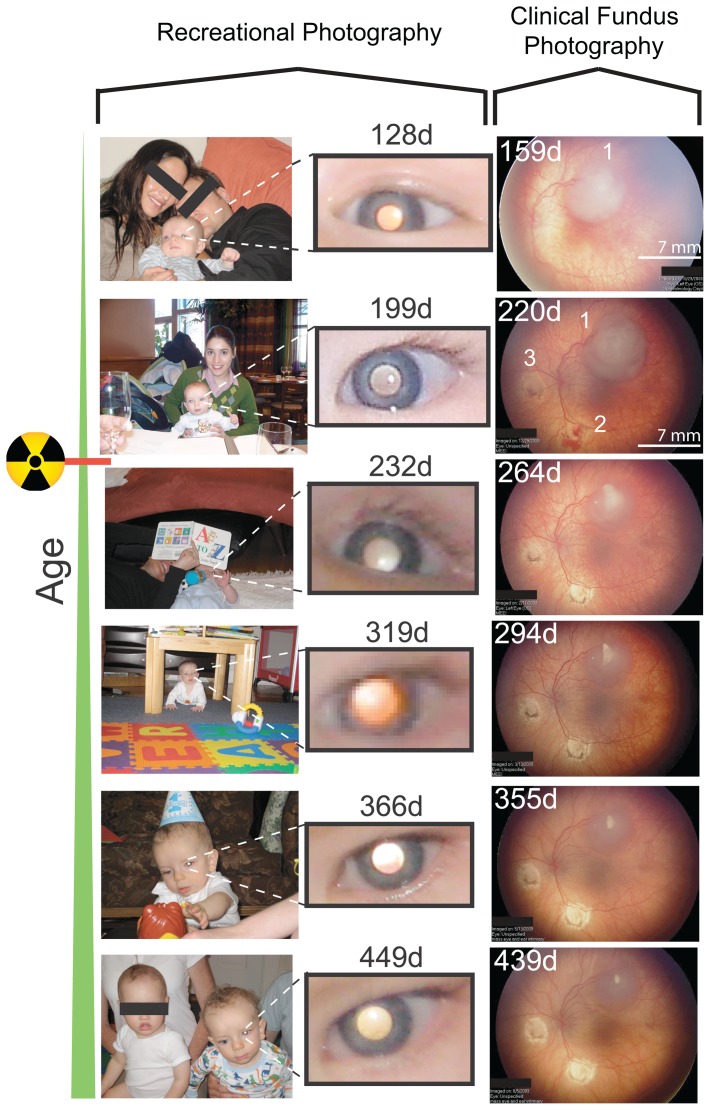
Longitudinal Set of Clinical Images of the Left Retina of Patient Zero Collected with Fundus Photography and Age-Matched Leukocoria in Left Pupil. The left retina contains three tumors; one large tumor at 12 o'clock, and two smaller tumors at 6 o'clock and 9 o'clock (the two smaller tumors were treated with laser photoablation therapy which resulted in tumor eradication and exposure of the sclera). The radiation symbol denotes the point in time when proton beam radiation therapy was administered to the left eye (age of patient is listed in days).

Because the right and left eyes of Patient Zero received different types of treatment (e.g., the right eye was not treated with proton beam radiation therapy, but was instead enucleated), it is possible that the colorimetric differences in right and left leukocoria are not caused by differences in tumor surface area or position, but instead are caused by changes in the surface properties of tumors (e.g., calcification) or retina that resulted from radiation or photoablation therapy. The calcification of the large tumor in the left eye, and laser photoablation of the two small tumors (at 6 o'clock and 9 o'clock), can be seen from clinical images of the left retina that were obtained with fundus photography (Figure 7).

**Figure 8:**
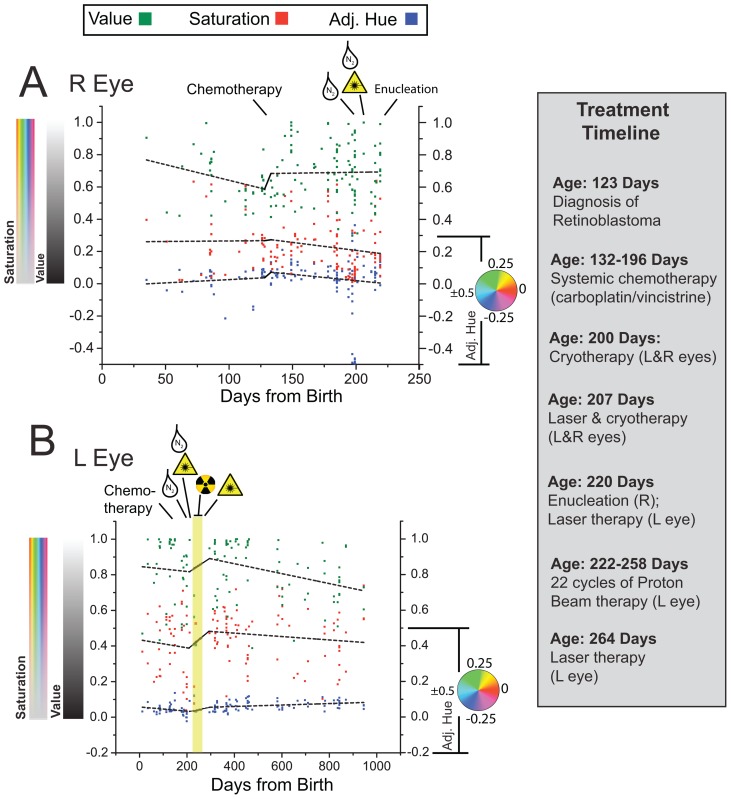
Longitudinal Plot of HSV Quantities of Leukocoria in Patient Zero. In order to project quantities of Hue in a Cartesian coordinate, we converted quantities of Hue to a linear scale. **A**) Plot of average HSV (per pixel) for leukocoric pupils from right eye. A linear fit of data points was made for two time periods: before and after systemic chemotherapy. **B**) Plot of average HSV (per pixel) for leukocoric pupils from left eye throughout the three-year period of photography. A linear fit of data points was made for two time periods: before the administering of proton beam radiation therapy, and after the completion of therapy (treatment timeline is listed at right panel).

In order to test the hypothesis that bilateral colorimetric differences are caused by treatment, we compared the longitudinal changes in the HSV properties of right and left leukocoric pupils over the three-year period of photography. First, we divided images into three longitudinal groups based upon the time of photography: (i) before treatment began (Period 1, age: 0–131 days), (ii) after chemotherapy, laser photoablation and cryotherapy (Period 2, age: 132–221 days), and (iii) after proton beam therapy and final treatment with laser photoablation therapy (Period 3, age: 259–945 days). In order to examine the variation of the HSV of each right and left leukocoric reflection, from day to day and throughout all three time periods, we plotted the HSV of each leukocoric pupil as a function of the patient's age (Figure 8). A linear fit was applied to the HSV data points for each treatment period in each eye (dashed lines in Figure 8).

The mean Saturation of left leukocoric pupils only varied from 0.400–0.459 (i.e., 13% variation) throughout all three periods of treatment (Table 2). The mean Saturation of the right leukocoric pupil from Periods 1 and 2 were 0.226 and 0.265 (i.e., 15% variation; p = 0.1606); the right eye was enucleated before the beginning of Period 3. Throughout all three periods of treatment, the mean Value of left leukocoric pupils varied only 0.822–0.835 (i.e., 2% variation); the mean Value of the right leukocoric pupil from Periods 1 and 2 were 0.641 and 0.696, respectively (i.e., 8% variation, p = 0.1407). The persistent difference in Saturation or Value of right and left leukocoric pupils throughout the entire three-year period suggests that the colorimetric differences between right and left leukocoric pupils are not caused by treatment, but are instead the result of differences in the surface area and/or the position of tumors in the right versus left eye.

**Table 2:** HSV Properties of Leukocoric Pupils in “Patient Zero” During Three Periods of Treatment.

Treatment Period	Left Eye			Right Eye		
	Hue^e^	Sat.	Value	Hue	Sat.	Value
**Period 1:** age: days 0–131 (n = 19 left, 37 right)a,b	16.5° (0.013°)	0.411 (0.145)	0.835 (0.194)	10.7° (0.156°)	0.265 (0.171)	0.641 (0.145)
**Period 2**: age: days 132–221 (n = 21 left, 109 right)a,b,c	13.9° (0.048°)	0.400 (0.195)	0.822 (0.170)	25.5° (0.244°)	0.226 (0.166)	0.696 (0.170)
**Period 3**: age: days 259–945 (n = 77 left)^c^	23.8° (0.024°)	0.459 (0.144)	0.823 (0.157)	n/a^d^	n/a^d^	n/a^d^

a For the right eye, a comparison of Periods 1 and 2 yielded p-values of p>0.05 for Hue, p = 0.1606 for Saturation, and p = 0.1407 for Value.

b For the left eye, a comparison of Periods 1 and 2 yielded p-values of p>0.05 for Hue, p = 0.7370 for Saturation, and p = 0.7622 for Value.

c For the left eye, a comparison of treatment Periods 2 and 3 yielded p-values of p<0.05* for Hue, p = 0.1484 for Saturation, and p = 0.9930 for Value. *Entries with asterisk indicate the given colorimetric property is statistically different between the two sets being compared at a 0.05 significance level.

d Treatment Period 3 was post-enucleation of the right eye.

* Entries with asterisk indicate the given colorimetric property is statistically different between the two sets being compared at a 0.05 significance level.

e For Hue, comparisons were made using the Wheeler-Watson test.

Error values in parentheses are standard deviation, except for those of Hue, which are circular standard deviation (CSD). CSD is a circular statistical analogue of standard deviation which measures the spread of the data points about the average center.

In conclusion, the colorimetric properties of the right and left eye of Patient Zero were different from each other before and after radiation therapy, and were generally stable over the three-year period of photography. The administering of proton beam therapy to the left eye cannot entirely explain the differences in the Saturation or Value of left and right leukocoric pupils. We also do not believe that the greater Value observed in the left eye arose from the exposure of sclera that resulted from photoablation of the two small tumors at 6 o'clock and 9 o'clock (see fundus photographs in Figure 7). For example, the Value (or Saturation) of leukocoric pupils did not change significantly as a result of laser photoablation therapy and exposure of the sclera (possibly because the bare sclera and tumor reflect similarly during flash photography). We conclude that the longitudinal stability of the colorimetric properties over the three-year period of photography is due to the stabilization of growth of the predominant tumor in each eye that was quickly accomplished for this patient by his early diagnosis at age 4 months.

### The Colorimetric Differences between Right and Left Leukocoric Pupils of Patient Zero Are Not Artifacts of Photography

Determining the exact exposure time and focal aperture (“f-number”) for each leukocoric image is necessary to determine whether the colorimetric differences that we detect between right and left leukocoric pupils of Patient Zero are the results of a photographic artifact, or are clinically relevant. For example, many of the leukocoric photographs of the patient did not contain *bilateral* leukocoria, which means that many of the right and left leukocoric pupils were contained in different photographs. It is thus possible that the colorimetric differences between right and left leukocoric reflections were caused – at least in part – by differences in the optical settings of the camera during the collection of each image (e.g., exposure time, focal aperture, flash mode, etc.).

The photographic settings for each photograph are embedded as EXIF data in each JPEG file, and can be viewed when the JPEG file is analyzed in software programs such as Picassa® (Google Inc., Mountain View, CA). The average time of exposure and average focal aperture were calculated for right and left leukocoric pupils, and were found to be statistically similar. The colorimetric differences between right and left eye are therefore clinically relevant. For example, the average time of exposure (t_exp_) of the 120 photographs with left leukocoric pupils was t_exp_ = 16.9±4.1 msec, *versus* t_exp_  = 18.6±4.1 msec for the 146 photographs with a right leukocoric pupil. Likewise, the average focal apertures (f) were similar: f = 3.7±1.0 for photographs with left leukocoric pupils; f = 3.9±1.4 for photographs with right leukocoric pupils. The EXIF data also documented that a flash pulse (from the xenon flash tube) was emitted during the collection of every leukocoric picture.

To ensure that the cropping of leukocoric pupils in this study is reproducible, we had two different researchers (AA and BT) crop the entire set of photographs and quantify the pupils in HSV color space (Table 3). The mean colorimetric properties of both eyes for Patient Zero and all controls are statistically similar (p>0.05) regardless of which researcher performed the analyses (denoted as “Trial 1” and “Trial 2” in Table 3). This similarity illustrates that colorimetric variations in leukocoric and non-leukocoric pupils are not the artifacts caused by variations in the practice of pupil cropping.

**Table 3:** Mean Colorimetric Properties Calculated From Images Containing Leukocoric and Healthy Control Pupils and Cropped by Two Different Researchers (Trials).

Eye	Hue^a^ (Trial 1)^c^	Hue (Trial 2) c	Sat.^b^ (Trial 1)	Sat. (Trial 2)	Sat. % dev d	Value b (Trial 1)	Value (Trial 2)	Value % dev
R (Lk^e^)	21.1° (0.215°)	17.7° (0.189°)	0.234 (0.166)	0.266 (0.188)	12.03	0.677 (0.1658)	0.665 (0.166)	1.77
L (Lk)	21.0° (0.033°)	20.7° (0.034°)	0.436 (0.159)	0.438 (0.156)	0.46	0.818 (0.168)	0.808 (0.174)	1.22
R&L (Lk)	21.1° (0.035°)	19.0° (0.109°)	0.325 (0.191)	0.344 (0.194)	5.52	0.741 (0.181)	0.729 (0.184)	1.62
R&L (Control)	347° (0.978°)	348° (0.647°)	0.319 (0.204)	0.318 (0.203)	0.31	0.288 (0.212)	0.267 (0.196)	7.29

a For each entry of Hue, the number in parentheses is the circular standard deviation (CSD) of the crops in that data set. CSD is a circular statistical analogue of standard deviation which measures the spread of the data points about the average center.

b For each entry of Value and Saturation, the number in parentheses is the standard deviation of the crops in that data set.

c Trial 1 was cropped by Brandon W. Taylor and Trial 2 by Alireza Abdolvahabi.

d Percent deviations (% dev) are calculated using: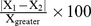.

e Lk: Leukocoric.

### Leukocoria Can Occur in Spite of “Red Eye Reduction” Technology

There is growing concern among clinicians that Red Eye Reduction and Red Eye Removal technologies will inhibit the ability of a digital camera to detect leukocoria [45], and possibly cause significant delays in the diagnosis of Rb. We point out that the two cameras used to collect photographs of Patient Zero were equipped with optional “Red Eye Removal” and “Red Eye Reduction” technologies (which are two entirely different types of technology), however, these technologies were not generally used by parents in this study. For example, the parents of Patient Zero utilized Red Eye Reduction flash mode in only ∼5% of the leukocoric pictures they collected (as determined by analysis of the EXIF data for each JPEG image), and the parents did not edit any of the images with Red Eye Removal software. Red Eye Reduction technology employs a flash with a series of two light pulses: the first pulse is intended to contract the pupil (immediately prior to the collection of the image), and the second pulse provides lighting during exposure. In contrast, Red Eye Removal technology is a software feature that edits a photograph (i.e., removes red eye) after it is collected. We conclude that the Red Eye Reduction feature did not entirely inhibit leukocoria in Patient Zero, possibly because the centrally located tumors blocked light from bombarding the retina, and inhibited the contraction of the pupil during the first flash pulse.

### Occurrence of Clinically Irrelevant “Pseudo-Leukocoria” During Flash Photography

In contrast to the possibility that Red Eye Reduction technology might inhibit the occurrence of leukocoria, there is also evidence suggesting that new models of digital cameras (such as the camera embedded within the Apple iPhone®) are causing clinically *irrelevant* leukocoria to occur in healthy children and adults (who do not have any known eye disease) at a higher rate than previous models of digital cameras. The cause(s) of this alarming increase in pseudo-leukocoria – alarming because it might cause parents to begin to overlook leukocoria that *is* clinically relevant – is not known. We hypothesize that this increase in pseudo-leukocoria is caused by: (i) errors in post-processing of the image after collection (i.e., “Red Eye Removal”), (ii) the type of “flash” or light source (i.e., a Light Emitting Diode (LED) in newer cameras such as the iPhone® *vs*. a xenon flash tube in older cameras), and/or (iii) the proximity of the light source to the lens (a general rule of thumb in photography is that retinal reflections are minimized by moving the flash source away from the camera lens, which is of course impossible in compact cameras).

Nevertheless, the leukocoric reflections that we detect in this study are clinically relevant, that is, are not likely to be caused by reflection of the optic nerve [32], or artifacts of advanced camera technologies. This conclusion is based on: (i) the absence of leukocoria in 305 images of pupils from 19 healthy control subjects, and the absence of leukocoria in adults that were photographed alongside each patient (data not shown), (ii) the correlation between the longitudinal frequency of leukocoria in Patient Zero and the progression/remission of disease, and (iii) the bilateral correlation between the lateral frequency and intensity of leukocoria and the clinical severity of each eye (Figure 4–5).

**Figure 9:**
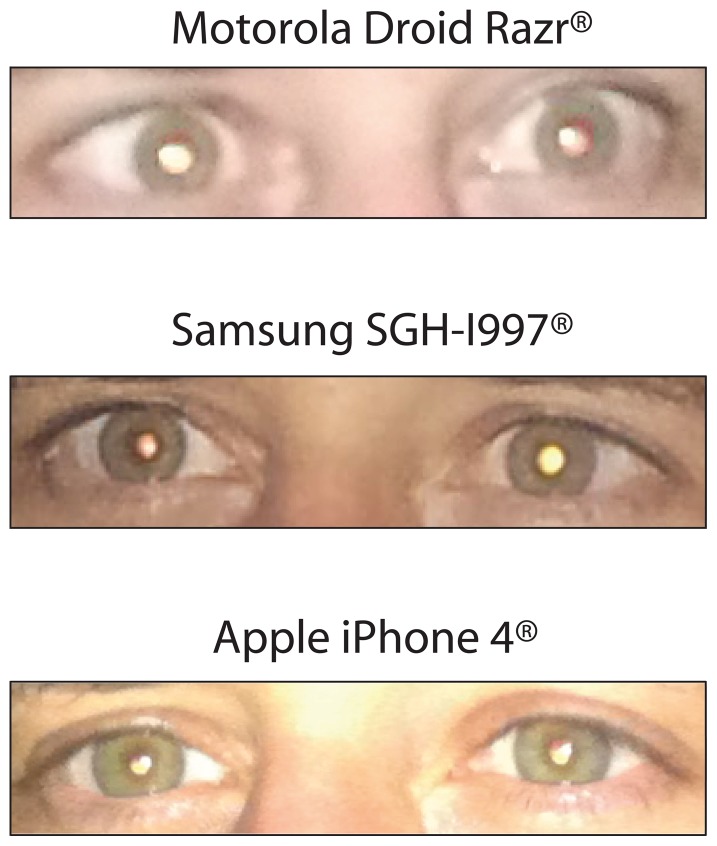
Examples of “Pseudo-Leukocoria” (Bilateral or Unilateral) in a Healthy Adult without any Known Eye Disease. Upper panel: photograph collected with a Motorola Droid Razr® and “pseudo-leukocoria” which can be seen in the right eye (unilateral). Middle panel: photograph collected with a Samsung SGH-I997® and with “pseudo-leukocoria” observable in the left eye (unilateral). Lower panel: photograph collected with an Apple iPhone 4® and “pseudo-leukocoria” can be partially seen in both eyes (bilateral). As described in the text, all photographs were taken in the same low-light conditions i.e., intensity of 0.0259±0.0074 µE/m^2^/s.

**Figure 10:**
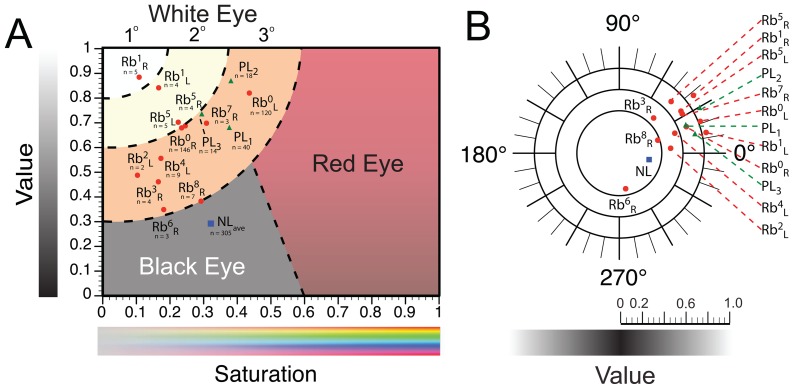
Saturation-Value Scale for Quantifying Leukocoria in Photographs of Children with Retinoblastoma. **A)** Sectioning the Saturation-Value plane of HSV color space into a useful scale for classifying pupillary reflexes in recreational photographs. In this proposed scale, leukocoria is divided into differing degrees of brightness and color concentration (1° being the brightest, least colored; 3° is the least bright and most colored); areas that likely represent a typical “red” or “black” pupillary reflex are indicated. Each data point labeled “Rb” refers to the average H, S, or V of all leukocoric images of one of nine patients; the superscript of each label refers to the patient number (beginning with zero); subscript text refers to right or left pupil. “PL” refers to Pseudo-Leukocoria from images of a healthy individual that were collected with one of three different camera phones; the subscript refers to the camera that was used to photograph the individual (see text). “NL” refers to Non-Leukocoric controls (average of right and left pupils) from healthy children (i.e., data contained in Figure 5 and Table 1). The value “n” below each Rb, NL, and PL point refers to the number of pictures from which each average was calculated. **B)** Plot showing the average Hue of cropped pupils from panel A.

In order to determine if pseudo-leukocoria can be easily distinguished (colorimetrically) from Rb-linked leukocoria, we collected and analyzed 72 pseudo-leukocoric images (of a healthy adult male) using three different digital camera phones equipped with a flash or light source (Figures 9 and 10; see Methods for more details on the type of cameras used). We determined that pseudo-leukocoria only occurred under low-light conditions, i.e., at 0.0259±0.0074 µE/m^2^/s (according to measurement with a digital light meter). This low-light setting was established by simply turning off overhead cool fluorescent lights in a windowed office. We found that “pseudo-leukocoria” did not occur during flash photography in the same room when the lights were turned on i.e., at 10.4±0.0074 µE/m^2^/s in the presence of a higher light intensity. The average colorimetric quantities of pseudo-leukocoric reflections (denoted “PL” in Figure 10) from all three cameras were grouped in similar color space (i.e., Hue: 15°–30°; Saturation: 0.3–0.4; and Value: 0.9–0.7), and were similar to the HSV properties of some Rb patients with 3° leukocoria (Figure 10). The similarity between Rb-linked leukocoria and pseudo-leukocoria suggests that any type of leukocoria detection software that might be engineered to alert users of digital cameras to the presence of leukocoria will need to discriminate between pseudo-leukocoria and Rb-linked leukocoria based (in part) on the higher rate at which leukocoria is likely to occur during the photography of a child with Rb compared to a healthy subject.

### Colorimetric Analysis of Leukocoric Photographs from Eight Additional Patients with Rb

As the first step in determining the range of HSV coordinates that will generally describe Rb-linked leukocoria in recreational photography, we plotted the Hue, Value, and Saturation of leukocoric pupils for all nine patients in this study (Figure 10). As shown in Figure 10, the average Value and Saturation of leukocoric pupils from each patient are Value >0.3, and Saturation <0.6. The leukocoria in all but one of the patients was characterized by a red-yellow hue.

In order to begin to establish a quantitative scale of leukocoria and a means of interpreting the colorimetric properties of pupillary reflexes in digital photographs, we sectioned the Saturation-Value plane of HSV color space into five regions (Figure 10): (i) 1° leukocoria, (ii) 2° leukocoria, (iii) 3° leukocoria, (iv) “black” eye, and (v) “red” eye. The exact boundaries of the regions that we propose in this scale of leukocoria are by no means definitive, and will likely change (slightly) as we analyze more photographs from more patients. In this particular scale, first degree leukocoria represents the most intense level of leukocoria, i.e., the brightest, least colored leukocoria. We therefore present this scale as a tentative first step in developing methods for quantitatively interpreting leukocoria.

### Improving the Timing of Diagnosis of Retinoblastoma in Developing Nations with Digital Photography

Over the past decade, the timing of diagnosis of Rb in underdeveloped countries has been improved by campaigns that increase the public awareness of Rb, and facilitate referral of children who might have Rb [13], [46]. The growing prevalence of digital cameras in developing nations (e.g., in India, which is predicted to have a compounded annual growth rate (CAGR) of ∼27% in its camera market in the next five years, outpacing the market growth in the USA) [47]–[49], and their growing use in telemedicine [50], [51] suggest that digital photography can play an increasingly large role in these types of ongoing Rb campaigns. We find it reasonable to predict that access to digital photographic devices will continue to increase for many families in developing nations [49] at a faster rate than access to pediatric clinicians who can screen for Rb with conventional methods. The photography of children by *parents* in resource-limited settings might, therefore, represent a rapid, economical, and effective method for decreasing the age of diagnosis of Rb in these environments.

### Can the Digital Camera Help Preserve Vision of Rb Patients in Highly Developed Nations?

Diagnostic challenges also continue to exist in highly developed nations, despite the high rate of survival. Improving the timing of diagnosis in developed nations will not lead to enormous increases in the (already high) survival rate, however, removing delays in diagnosis can result in greater degrees of vision preservation [14]–[18]. Recent reports have described great deficiencies in the ability of pediatricians to detect Rb, possibly due to inconsistencies in the administering of the “red reflex” test, or simply because a child only receives (typically) ∼12 examinations by a pediatrician during the first two years of life [52]. Moreover, regular examinations of a child's vision – which might also detect Rb – typically begin after the age of 3 years, which is outside the typical age of diagnosis [52]. In contrast to this limited number of pediatric examinations, a child who lives in a highly developed nation will be photographed hundreds or thousands of times by parents, guardians, relatives, or acquaintances during the first two years of life.

We point out that although many individuals own digital cameras in developed nations, a significant portion do not, and their increasing access to digital photography over time might lead to improvements in the use of digital photography to detect Rb. For example, it is estimated that ∼10% of individuals in the USA do not have access to a digital camera (either as a standalone camera or a camera phone [53], [54]).

The use of the digital cameras to detect intraocular abnormalities might represent the most economical and rapid method for improving Rb diagnosis in developed nations. The potential of digital photography cannot be overestimated, in our opinion, because the detection limits of digital photography have not even been established, and might be much higher than currently appreciated. For example, digital photography appears to be able to detect early stage Rb that presents with a “gray” pupil (Figure 3) – which might not seem abnormal to a parent or clinician – prior to the presentation of classic “white” (or yellow) leukocoria. Utilizing the full potential of digital photography in screening Rb will likely require the development of a computer software that can alert the photographer (or viewer of the image) to the presence of an abnormal pupillary reflection that might or might not be obvious to the naked eye.

## Conclusion

The primary *clinical* result of this study suggests that a leukocoric photograph of a child with Rb can provide more information than a binary readout of leukocoria. We have shown that the quantity of Saturation and Value of a leukocoric pupillary reflection (in HSV color space) might be a crude metric for approximating the degree of leukocoria, which might be – for tumors in certain positions – a convenient expression of the total reflective surface area of intraocular Rb tumors.

This study also shows that “low frequency” leukocoria can be overlooked more easily by parents than “high frequency” leukocoria. For example, the parents of Patient Zero did not notice leukocoria until it appeared in >60% of pictures per day, and >10% of pictures per month; in fact, approximately 3 months elapsed from the time leukocoria emerged to the time it had increased to sufficient frequency that parents began to notice leukocoria. Increasing public awareness about leukocoria can accelerate diagnosis by preventing parents from overlooking sporadic leukocoria during the early stages of Rb. For example, although “Patient Zero” was diagnosed 5–8 months earlier than the average age of diagnosis for bilateral Rb [16], [55], it is reasonable to predict that an earlier diagnosis at 12 or 35 days old – when the leukocoria first emerged – would have improved the patient's outcome.

This study only examined photographs of 9 patients with Rb (and only a single patient in longitudinal and bilateral detail), however, we suspect that the primary clinical finding of this study – that distinct ocular sets of Rb tumors produce distinct colorimetric patterns during amateur photography – will be found to be generally applicable, to some degree, with leukocoric photographs of other children with Rb. This zeroth order approximation is based on the assumption that: (i) the possible position of Rb tumors, in both time and space, is quite narrow (i.e., the surface area of a child's retina is <11 cm^2^, and Rb tumors typically form before the age of 5 years), and (ii) the photography of children by parents occurs at multiple angles, which will increase the probability that light will bombard tumors in both central or peripheral positions. We believe that the optical diversity of recreational photography – the collection of images at different angles and lighting conditions – by no means lowers its utility in Rb detection, but actually improves its applicability by increasing the probability that leukocoria will be eventually observed, regardless of the tumor position.

Analyzing additional libraries of photographs – similar in size to the library of Patient Zero – from more Rb patients will be necessary to determine whether the colorimetric properties of leukocoria have general clinical relevance, and to fully interpret the clinical implications of HSV quantities of a leukocoric image. The software that we used to analyze photographs (Adobe Photoshop®) is readily available, and the algorithm for converting RGB to HSV color space is operable in Microsoft Excel®. Researchers or clinicians without expertise in computer science should, therefore, be able to carry out the colorimetric analyses that we describe on photographs of other patients. Collecting a database of HSV coordinates of leukocoria from other patients will help establish a quantitative definition and scale of leukocoria, which might prove useful for quickly approximating the clinical severity of Rb when a parent reports leukocoria.

The digital photography of children in recreational settings is by no means as useful as high-resolution clinical methods for examining and imaging the retina (e.g., ophthalmoscopy and fundus photography). This technical disparity notwithstanding, the high frequency of photography of children by parents throughout the entire five year period of Rb susceptibility, combined with the growing prevalence of digital photography, is resulting in the accumulation of enormous, longitudinal sets of images that represent crude retinal scans. The colorimetric analysis of these types of large photographic libraries – over 7,000 images for the single patient in this study – might be, as an aggregate, useful for screening or assessing Rb. We envision that the creation of computer software that can automatically detect and quantify leukocoria – within thousands of images from a parent's library of “baby pictures” or during photography or web-based social networking – will facilitate the automated and instantaneous screening of leukocoria in children throughout the entire period of their Rb susceptibility.
